# ‘Dented’ and ‘Resuscitated’ masculinities: The impact of HIV diagnosis and/or enrolment on antiretroviral treatment on masculine identities in rural eastern Uganda

**DOI:** 10.1080/17290376.2014.986516

**Published:** 2014-12-02

**Authors:** Godfrey E. Siu, Daniel Wight, Janet Seeley

**Affiliations:** ^a^ PhD, is a Senior Scientist at the MRC/UVRI Uganda Research Unit on AIDS, Entebbe. P.O Box, 49, Entebbe, Uganda; ^b^ Lectures in the Child Health and Development Centre, College of Health Sciences, Makerere University, P.O Box, Kampala6717, Uganda; ^c^ PhD, is Professor and leads the Children, Young people, Families and Health Programme in the MRC Social & Public Health Sciences Unit, 4 Lilybank Gardens, GlasgowG12 8RZ, United Kingdom; ^d^ PhD, is Professor in the School of International Development, University of East Anglia, NorwichNR4 7TJ, United Kingdom; ^e^ PhD, is Professor in the London School of Hygiene and Tropical Medicine, Keppel Street, LondonWC1E7HT, United Kingdom; ^f^ Heads the Social Science Programme in the MRC/UVRI Uganda Research Unit on AIDS, Entebbe. P.O Box, 49, Entebbe, Uganda

**Keywords:** HIV, antiretroviral treatment, resuscitated masculinity, dented masculinity, Uganda, VIH, les traitements antirétroviraux, la masculinité ressuscitée, l'image ternie de la masculinité, Ouganda

## Abstract

There is limited research on the impact of HIV or its treatment on men's identity construction and gender roles in sub-Saharan Africa. Based on in-depth research with 26 men in rural Uganda, this article discusses men's vulnerabilities and shifting gender relations and sense of masculinity resulting from HIV infection or enrolment on treatment in eastern Uganda. The findings suggest two broad categories of masculinity: respectable and reputational. HIV infection and illness dented masculinity as men lost authority within the domestic sphere. A weakened provider role and over-reliance on wives and children undermined masculinity as family head, and social sanctioning of their sexual activity, undermined conventional masculine identities predicted on reputation. However, treatment led to a more reflexive approach to demonstrating masculinity, increased attentiveness to health and restored hope to father children free of HIV, resuscitating respectable masculinities. The balance between eroded and restored masculinity varied between men by their treatment history, age, family composition and state of health. HIV support agencies need to pay attention to the way HIV and antiretroviral treatment (ART) influence men's perception of their masculinity and support them to overcome the anxieties about dented or eroded masculinity, while building on the positive ways in which treatment restores masculinity to support men's adherence to HIV treatment. In particular, there is a need to support men's engagement in productive activities that bring income so that men can regain their provider roles following ART and restore their respectability in both the public and the domestic sphere.

## Résumé

On a effectué peu de recherches sur l'impact du VIH ou de son traitement sur la construction identitaire des hommes et les rôles masculins et féminins en Afrique subsaharienne. Tout en s'appuyant sur une enquête approfondie auprès de 26 hommes en milieu rural ougandais, cette étude examine les vulnérabilités, le changement de relations hommes-femmes et la perception de masculinité chez les hommes infectés au VIH et subissant le traitement dans la région orientale de l'Ouganda. D'après les résultats, il existe deux principales catégories de la masculinité : le respect et la réputation. L'infection au VIH a terni l'image de la masculinité parce que les hommes ont perdu leur autorité au niveau familial. Un rôle affaibli de subvenir aux besoins de la famille et le recours excessif aux femmes et enfants ont sérieusement affecté l'image de la masculinité en tant que chef de famille. L'activité sexuelle et l'identité masculine fondée sur la réputation ont été également affaiblies. Pourtant, le traitement a entrainé une approche plus réflexive de la masculinité : une plus grande attention accordée à la santé et l'espoir d'engendrer les enfants sans VIH voire la ressuscitation de la masculinité respectable. La masculinité gravement affaiblie et celle regagnée ont varié entre les hommes en fonction des critères suivants : antécédents de traitement, âge, composition de famille et état de la santé. Il faudrait que les organismes de soutien aux personnes vivant avec le VIH prêtent une attention particulière à l'influence du VIH et les traitements antirétroviraux sur la perception des hommes en matière de leur masculinité. Ces services devraient également appuyer ces hommes de surmonter leurs inquiétudes par rapport à la réputation masculine entachée ou gravement affaiblie tout en soulignant les modes positifs du traitement tels que l'observance du traitement du VIH qui leur permettent de regagner leur santé et masculinité. Il est nécessaire notamment d'appuyer la participation des hommes aux activités génératrices de revenus pour qu'ils puissent regagner la confiance en jouant leurs rôles de subvenir aux besoins familiaux tout en suivant les traitements antirétroviraux. Ils pourraient donc regagner le respect aux sphères domestique et public.

## Introduction

HIV and AIDS remain a major public health problem in sub-Saharan Africa (SSA), and especially in Uganda where both prevalence and incidence have increased in the past five years. Recent trends indicate that the annual HIV incidence in Uganda increased from about 115,000 new infections in 2007 to over 130,000 in 2013 (Republic of Uganda 2014). As a consequence of rising incidence and greater longevity of people living with HIV due to treatment, HIV prevalence in Uganda has risen from 6.4% in 2005 to 7.3% in 2011 (Ministry of Health (MoH) Uganda *et al*. 2012), with about 1.5 million people currently estimated to be living with HIV (Republic of Uganda 2014).

As elsewhere in SSA, the provision of free antiretroviral treatment (ART) now forms a key policy component of Uganda's national response to the HIV epidemic. This favourable policy framework has led to a rapid increase in the number of people accessing AIDS treatment in recent years. By the end of 2013, there were about 570,000 people with HIV in Uganda receiving ART, with females outnumbering men at the ratio of 2:1 (349,068 females compared to 174,582 males) (Republic of Uganda 2014). When used early and consistently, ART can decrease the rate of HIV transmission (Attia, Egger, Muller, Zwahlen & Low 2009; Castilla, Del Romero, Hernando, Marincovich, García & Rodríguez 2005) and can delay the progression of HIV to AIDS and prolong life, shifting the HIV-related disease from an acute terminal illness to a chronic long-term illness (Colvin 2011; Reynolds 2011).

Research, largely from higher income countries, on men's experience of living with chronic illnesses such as depression (Emslie, Ridge, Ziebland & Hunt 2006; Emslie, Ridge, Ziebland & Hunt 2007), hypertension (Emslie and Hunt 2009), prostate cancer (Oliffe 2006; Oliffe 2007; O'Brien, Hunt & Hart 2005; O'Brien Hart & Hunt 2007) and breast cancer (Pituskin, Williams, Au & Martin-Mcdonald 2007) suggests that chronic illnesses can threaten the construction of an idealised masculine identity. Identity dilemmas result from losing loved attributes, physical functions, social roles and personal pursuits (Charmaz 1990; Charmaz 1994), and this greatly impacts on people's response to illness, including seeking treatment and coping (Ezzy 2000; Gannon, Glover, O'neill & Emberton 2004). To date, with the exception of Sakhumzi (2008), there has been limited research in SSA on how living with HIV as a chronic illness affects masculinity. In South Africa, HIV diagnosis undermined masculinity because the illness made it especially difficult for men to fulfil their provider and sexual roles (Sakhumzi 2008). Sakhumzi concluded that ‘ … an HIV diagnosis resulted in incongruence between what men had internalised as definitive features of masculinity and their post-diagnosis material and bodily conditions’ (p.56).

We conceptualised masculinity as the social and cultural expression of what it means to be a man (Kimmel 1987). Theories of gender and masculinity suggest that gender attributes and roles are allocated according sex (male or female biology) but are acquired through distinguishing oneself from the complementary but opposing identity (Brittan 1989). In most societies, masculinity is often expressed through aspiring to have multiple sexual partners, being physically and emotionally robust, risk taking, independence, being a family provider, dominant decision-maker and subordinating and controlling women and children (Barker & Ricardo 2005; Jewkes & Morrell 2010; WHO 2007). However, because there is diversity in contexts and experiences upon which notions of masculinity are constructed, the meaning of masculinity may vary from society to society, and so there are multiple masculinities (Kimmel 2000). In the theory of hegemonic masculinity, Connell argued that in every society there is usually a culturally dominant ideal of masculinity, against which all other masculinities are measured (Connell 2005). The notion of hegemony implies that there are orthodox or conventional ideals endorsed and aspired to by all men which can be distinguished from ‘other’ or ‘subordinate’ masculinities (Connell & Messerschmidt 2005). In this article we seek to extend this literature by examining men's vulnerabilities and shifting gender relations and sense of masculinity resulting from HIV acquisition, AIDS illness and subsequent treatment in eastern Uganda. Specifically, the article analyses how the experience of HIV and AIDS, and/or ART modifies masculine identity and men's gendered roles within the household and society.

## Ethical considerations

The study was reviewed by the Science and Ethics committees of The Uganda Virus Research Institute and The University of Glasgow Faculty of Social Science, and cleared by the Uganda National Council for Science and Technology. Informed consent was obtained from all interviewees, and pseudonyms are used for both the study village and interviewees to enhance anonymity.

## Study setting, design and methods

This paper is based on ethnographic data collected from Mam-Kiror Village, in Busia District, about 200 km south east of Kampala, Uganda, between August 2009 and August 2010. Ethnographic research, which relies heavily on participant observation, spending an extended period of time in the field and flexible application of interviews, can provide more valid data than structured interviews, group discussions or questionnaire surveys. This is because in ethnography the researcher is not creating an artificial research environment but is observing interactions that would probably be happening anyway, implying that the data are less subject to social desirability bias (Goodson & Vassar 2011).

At the time of data collection the study village had a population of about 750 people, the majority of whom were Iteso. By 2005 Busia had a higher HIV prevalence (10%) than the national (6.4%) average. The main source of cash for men was artisanal gold mining and trading, while the majority of women carried out crop cultivation. The first author (GES) conducted in-depth interviews with 26 men: nine receiving free HIV treatment from a public facility, eight who had dropped out or had not initiated treatment despite testing, six who suspected HIV infection but had not sought testing and three men who had other health concerns unrelated to HIV, included primarily to provide comparative data and mask the focus on men infected with HIV to minimise their stigmatisation.

The interviewees were selected through purposeful and snowball sampling methods. A total of 12 participants (nine receiving HIV treatment and three who tested but did not seek treatment) were accessed with the help of their treatment providers. The remaining participants were selected through snowballing. This was facilitated by two of the participants receiving HIV treatment who knew others in the village with similar health complaints but had not tested or who had dropped out of HIV treatment. Snowball sampling helps to reach hidden populations since the required sample is accessed through a referral process by other participants already taking part in the study (Denscombe 2010). Potential participants were briefed about the study by their peer, and if they indicated willingness to participate, they were referred to the researcher for further information and inclusion in the study.

The interviews were based on a flexible topic guide, which included topics such as ‘what it means to be a man’, ‘experiences with HIV and AIDS’ and ‘how HIV infection, HIV-related illness, and treatment affect domestic and societal roles, relationships and family situation’. Interviews were audio recorded and transcribed. The interviews were complemented with participant observation. GES lived in the village for a year and interacted with local people, including the 26 men interviewed, listening to and sharing their day-to-day stories and conversations about social life, masculinity and health. Some conversations were with men alone, but often they included family members, neighbours, peers or colleagues. Interactions took place in different contexts and sites within the village including homes, work places, bars, restaurants, social gatherings or during a walk, and field notes were usually written at the end of each day. While some interviewees may have not felt free to discuss intimate issues and therefore may have portrayed themselves differently, as a way to construct an image they perceived would be socially desirable, the participant observation and the long time GES spent interacting with different people as a native interviewer in the study area assured that reliable data were collected for triangulation. In addition, the other co-authors (JS and DW), who are both from the UK, provided external insights, enabling a reflective analytic space.

## Data management and analysis

We used the framework analysis approach (Ritchie & Lewis 2003) and managed the data using Nvivo 8. The interviews were transcribed and translated into English by an experienced translator and the first author (GES). GES then read the 26 transcripts, and a selection of observation notes, to identify preliminary thematic categories for a coding schedule. The coding framework was designed by all authors based on themes explicit in the data plus emergent categories. Systematic coding was then undertaken by GES. Themes were summarised in a matrix, and explored for recurrence, commonalities, variations and relationships. In presenting the results of this study, both the participants and the village of study are given pseudonyms.

## Findings

### Interviewees’ characteristics

The interviewees were aged between 27 and 51 years (median age 39), and most had less than seven years of schooling. All except three had children of their own, four were separated from their wives and one was widowed. Most had multiple livelihood sources but generally earned small incomes. Although when interviewed only 10 reported being actively involved in artisanal gold mining, the dominant occupation of men in the study area, all had been involved in mining at some stage in their life. Seven men had stopped mining or business due to illness.

### Two case studies

To illustrate the social experiences of living with HIV and/or ART as a man, we present two case studies: Jeremiah and Isaac. Their stories underline two core themes around masculinity: dented and resuscitated masculinity. The English word ‘dented’, from which we derive the concept dented masculinity, refers to a shallow deformation on the surface of an object resulting from an impact. This metaphor is suggested to open up particular analytical possibilities about the relationship between HIV diagnosis and changes in masculinity as experienced by men. Broadly, dented masculinity can be thought of as the negative changes and sense of loss to one's masculinity suffered as a result of HIV acquisition, HIV-related illnesses and/or enrolment on ART. Resuscitated masculinity refers to the sense or real experience of revival or sustenance of one's masculinity following HIV diagnosis, HIV-related illness and/ or ART.

#### Jeremiah: dented masculinity

Jeremiah, aged 40, had been on ART for about a year and a half, but looked frail throughout the fieldwork. Prior to illness, Jeremiah was one of the relatively successful artisan gold miners in the village. He had four years of education, and four children aged between 2 and 14 years, born to 2 of his 3 wives. By the start of fieldwork in 2009, all his wives had separated from him. Jeremiah's first wife had separated about a year earlier, resenting his decision to marry other women, although she had tolerated them for some time, while the other two had left him within three months following the onset of his repeated ill-health. On the insistence of Jeremiah, his first wife had left behind her two sons, aged 10 and 12 years old. Most times these young boys cared for themselves and their ill father, but sometimes Jeremiah's old mother helped with cooking. Although in February 2010 Jeremiah's first wife returned because she ‘sympathised’ with him and wanted to take care of her children, she did not stay at home consistently, frequently going to work in a bar for several weeks without her husband's consent.

Following Jeremiah's illness, which involved being bedridden for two years, and reduced earning, his wives became increasingly defiant and disrespectful of him. He reported failing to challenge one wife's revelation that he was not the father of her two-year-old child because he was too weak physically. Jeremiah reported a loss of status as head of family, and a reversal of power between himself and his wives. He was especially disturbed that his first wife, who had returned, often went to drink till late in the night with other men and in complete disregard of his feelings and criticisms. Jeremiah blamed this on his lack of physical strength to prevent his wife from going out, and also his fear to antagonise her lest she left again.

Jeremiah's illness also isolated him from male peers, which meant that many opportunities, including those to make money, had bypassed him. Jeremiah was, however, positive about the impact of treatment, and towards the end of fieldwork presented his masculinity as somewhat restored: ART would improve his health and in the process he would regain his self-worth. ‘[ … ] you wait when the body improves; you will soon see me at the trading centre with other men’.

#### Isaac: resuscitated masculinity

Isaac was 37 years old, had one child and like Jeremiah, had four years of education. He looked energetic and well built, evidence of his involvement in the highly physical artisanal gold mining work in the village. He had been on ART since 2004 when he tested HIV positive following recurrent illness. His third and only surviving wife had earlier been diagnosed with HIV through antenatal care, following three miscarriages and a still birth. Isaac's first two wives had reportedly died after separating with him and he reported that ‘on hindsight they might have died due to this disease [AIDS-related disease]’. Isaac described being confused about whether one of his wives infected him, and if so, which one, or whether his acknowledged risky sexual behaviours had infected them.

Prior to testing positive for HIV, Isaac's major concern was that at his age, he still had no children. His first two wives, who divorced him, had not borne him any children and he had hoped that his current wife would. However, as he put it, ‘ … but little did I know that it was this disease killing our children’. Following confirmation of his HIV seropositivity, and a still birth, public and family commentary about him began to grow for Isaac to ‘stop wasting time trying to have children’. Various people, including his own brothers would talk ill of Isaac saying: ‘this one with slim [AIDS] is also trying to produce children! Will they really become proper children?’ It was thought that people with HIV stood no chance of having healthy children. Isaac interpreted this as an insult to his masculinity and social status, reinforcing his determination to have a child. When he learnt that it was possible to have a child after ART, Isaac opted to ‘try again’, but this time after discussing his desires with his counsellor.

With ART, and having followed the counsellor's advice to take his wife to the hospital for delivery and to receive the prevention of mother-to-child transmission (PMTCT) service, Isaac's dream of having a child of his own, free from HIV, was realised. The birth of his daughter considerably reversed his family's and the public's perception of him, his wife and the treatment they were receiving. Many could not believe that the child was born without HIV, saying ‘it is just a made up story’. Following his success with ART, Isaac resolved to adhere to the recommendations of the counsellors, including that one does not have extramarital sexual relationships. Isaac said that, in hindsight, he believed extramarital sex is not the right way to live as a man as ‘ … it is dangerous to health  … so I am different now’. ‘He summed up the overall impact of ART on his life as  … although there are days that are different and you feel ‘downhearted’, I think it is better to just stay alive, try working and look after your family’.

### Masculinity before HIV and AIDS, and ART

Participants described various ideals a man was expected to demonstrate to be considered sufficiently masculine in Mam-Kiror. Although there was a shared understanding of masculinity, the differences between accounts suggested two variations which, following Wilson (1969), we call ‘respectable’ and ‘reputational’ masculinity. Respectable masculinity was endorsed by ‘the wider society’, which included not only primarily women but also men, in-laws and religious ministers. The key ideals in this values system were marriage, fathering children and providing for them, sexual fidelity and demonstration of wisdom and respect of self and others. Reputational masculinity was primarily endorsed by men amongst themselves, and included sexual achievement and fathering many children, physical strength, being powerful over women and children, socialising and ‘compulsory’ spending on leisure. However, there were ideals that were shared by both men and the wider society, within the reputational and respectable value systems, including a strong work ethic, financial independence, wealth, having money and being resolute (Siu, Seeley & Wight 2013).

### Masculinity after HIV infection and ART

Men's discussions of their experiences and identities as HIV and/or ART patients were framed around understandings of their current and potential social roles in their households and society, rather than reducing their circumstances to a mere medical problem. They repeatedly discussed their health condition from the perspective of husbands, fathers, brothers, sons-in-law, friends or as co-workers. The narratives revealed a diverse range of negative and positive experiences and a sense of self-worth and identity as men, suggesting that in some ways masculinities were dented, and in other ways they were resuscitated/restored by HIV or ART.

#### Dented masculinities

Participants’ accounts revealed that both HIV infection and ART negatively impacted on masculinity in multiple ways. While some men clearly distinguished between how HIV diagnosis and ART affected their masculinity, many did not. Rather, HIV and AIDS, and ART were discussed as one condition, perhaps because for many, ART was initiated immediately following their diagnosis with HIV.

#### HIV and wives: powerlessness, authority and control

Many men reported a degree of powerlessness and loss of control over members of their households, especially wives, following severe illness. For most men, the loss of physical strength, a resource they often relied on for enforcing orders by physically punishing insubordinate family members, was the main way in which masculine authority was lost in the home. As Jeremiah's account illustrates, he lost authority over his wife because he had grown physically weaker than her due to illness:

You see I cannot say much about her. She does so many things which I am not happy with, for example she can go to drink with other people and return late at night … she even goes for discos and returns in the morning. In my condition [illness/weak body] I am not able to stop her. I could not tell her  …  I do not know what is in her mind, so I feared*.*

The anxiety around authority in the household was mostly expressed by men who had been very ill prior to ART or those who, over time, grew weaker despite ART. They reported increased defiance of their orders and the undermining of their authority by their wives who had no fear of being assaulted or censured. In the extreme cases, some men feared physical violence from their wives, while some reported having repeatedly experienced verbal abuse. Some wives of men who were sicker or weaker confirmed this changing gendered power, maintaining that they had ceased to see their husbands as threatening as before. Moses’ (age 46 years) wife, for instance, admitted to a shift in power balance and authority between her and her husband, with her progressively becoming the more dominant person in the family. On a number of occasions she said ‘ … I am now stronger than him physically, so he cannot beat [or harass] me … ’ While she desired that her husband improved, she expressed relief that his aggressiveness towards her and the children had considerably reduced, compared to his former self. Thus, the various narratives revealed that HIV infection and/or AIDS not only resulted in considerably diminished strength, but also, for many men, the loss of use of violence as a means to enforce their wishes over other family members.

In general, participants’ accounts suggested that men living with HIV infection and/or ART, both younger and older, endured levels of mistreatment and oppression in their households in ways that substantially contradicted their expectation to enjoy the traditional masculine privilege of respect. They underwent varied but generally challenging experiences with regard to their expression of authority and power within the domestic sphere. The care they received and the relationships with their households were complex and largely disempowering.

#### Reliance on wives and children for care and provision

Men living with HIV described being over reliant on wives and children for care, particularly to be fed and washed, and to provide money for the family. Like Jeremiah, many men reported that their ability to provide for the family's needs, including school fees, medical care and inheritable wealth, had reduced substantially due to the illness. This meant that wives, as well as children, tried to fulfil these roles, especially when their husband/father was bedridden, which for most was frequent. With a seriously reduced provider role, some men talked about ‘losing a voice in the home’, greatly undermining their self-confidence as family heads.

The family provider role had given these men power and control over their dependents either by availing or withdrawing material support from them. However, a weakened provider role resulted with the onset of serious chronic illness, leading to a complete reversal of roles and influence for most men, since they had become dependent on their wives and children for care and support. Moses, for example, said that his children and wife often disregarded him and, he often ‘pleaded’ with them to do what he wished but none listened to him. Some fathers had to comply with the terms set by their children who earned an income, some of whom were as young as 10 years, because of what these children could provide for their care. These men also frequently reported that their HIV status had left them extremely vulnerable to being criticised by their wives at the slightest provocation. Some men stated that their women tended to express ill will through victimising and blaming them, leading them to feel guilty and disgraced. In the event of disagreements, some wives tended to remind them of their role in bringing the infection and at times told them to seek care or support from those women from whom they got infected, or even threatened to leave, although very few actually divorced. Like Jeremiah, whose wife evidently appeared to have control over him, Job, age 45 (not tested but suspected infection) also decried his growing ‘hopelessness’ before his wife who was ‘often up’ against him, verbally abusing him for his inability to provide, yet in the past she used to respect him:

 … eh eh, she no longer considers me important. You cannot say a word, I am just useless and yet when I was okay …  she married me because of the money I was making … oh, I was also a real man.

However, the degree of desperateness felt due to their inability to provide for their family varied with their health, but not, apparently, by age. Both younger and older men expressed similar concerns. Only one man (Leo, age 28, receiving septrin) argued that his ability to provide was never significantly altered by HIV as he was already ‘struggling’ to maintain a livelihood before HIV acquisition.

As their health improved due to treatment, many men described attempting to return to do hard physical work to earn income. However, most worried that strenuous work threatened the health gains from treatment, so they had to work less compared to other people. In addition the sympathies from work colleagues who knew about their HIV infection and/or treatment often reminded them that they were different, undermining their masculinity as hard workers.

#### ART and sexuality

The majority of participants revealed having experienced physical and emotional dysfunction, as well as social censure, with regard to affirming their sexuality following HIV diagnosis or ART. Although ART improved their health, resulting in some reporting that they had renewed interest and energy to engage in sex again, the majority expressed very little confidence in their abilities to make sexual advances or compete with other men for partners. Nearly all stated that adjusting to a normal sexual life with HIV or ART was a significant challenge and described sex with ART as ‘different’ or that ‘sex drive had reduced’, while others stated that, even in marriage, ‘sex had to be minimised’. For some, the main reason for reduced sexual intercourse with their wives or other partners was the feeling that they were less physically attractive and desirable, or that no new partner would accept them, given their weak physical appearance or renowned ‘bad’ sexual past. Jeremiah narrated his experience with his wife: ‘Now we do not [have sex]. What can I say to her! I just look at myself and I know I am not able, even she can say, “ … and you are sick”’. Other men frankly stated that being on ART made sex less emotionally appealing because swallowing ART drugs on a daily basis had a psychological effect of making them feel too ill, or their wives thought they were. Isaiah, age 49 (who eventually discontinued ART), described his experience:

With regard to those issues [sex], there was nothing much to talk about because you have to go slow. You see when I became sick, I was so sick that I was thought to be dying anytime, so now, when I look at myself I cannot even bother with that [sex]. Let me say that even with the drugs, I could not see my wife accepting me because she had already started to refuse. So in this condition, when you are not well like this, you do not even have agogong [morale/drive], emame etau [there is no interest]. But before we got this problem, everything was okay and there were no quarrels over that.

However, the narratives of men like Alfred (age 38), who were no longer confident about their sexual abilities, also seemed to mirror what they had been advised by their counsellors or by others with HIV: to abstain from sex and conserve energy. As Ben (age 36) described, ‘they say that when you are in this situation, one round [of sex] is like running 10 kilometres; it takes a lot of energy, and also we have to be careful not to catch another dangerous virus’. Some wives of the interviewed men also confirmed that ART treatment appeared to have affected their husbands’ sexual abilities. The wives of Abraham (age 50) as well as Noah (age 50), for example, acknowledged that their husbands had become relatively impotent following ART, but were quick to add that they were understanding and ready to adjust to their husbands’ capabilities. Abraham's wife described her husband's decreasing sexual abilities and how she had accepted to live with it as follows:

It [his sexual performance] has reduced … sometimes he apologises. But I also know that when you are on these drugs, they make you drowsy, so there is no need for me to be over-demanding …  So, on that one [sex], whatever he can manage is what I will accommodate.

Men's accounts also highlighted the stigmatisation of sexual activity by people known to be living with HIV. Irrespective of treatment, there were widespread negative perceptions of their sexuality, relationships and reproductive behaviour, with widespread gossip, and sometimes direct mocking, especially if the man in question was relating with a non-marital partner. As a result, unlike men with unknown HIV status who might discuss their relationships with their peers, men with HIV could not discuss them even with peers for fear of being criticised, which undermined their expression of masculinity through sexuality and reproduction. Isaiah (age 49, dropped ART), during one conversation, described this experience as ‘being driven underground and hiding all the time from people as if you are not a man’. It was therefore less surprising that with the exception of Ben (age 36) and Mike (age 32) who remarried, all other interviewees living with HIV reported neither initiating new sexual relationships nor having attempted to re-establish old relationships outside marriage ever since their HIV diagnosis. However, there may have been underreporting of their sexual relationships since, during his fieldwork, GES became aware of at least three non-interviewees in the village known to be on HIV treatment who were alleged to be involved in non-marital sexual relationships, and some interviewees had fathered children with those women. The two men who remarried and who had reportedly initiated new relationships after HIV diagnosis were relatively young compared to the majority of the interviewees who emphasised their lack of interest in extramarital sex, suggesting that age may have an important influence in the pattern of sexual activity following HIV diagnosis/ART.

There was a great sense of guilt in being seen to have infected one's wife with HIV or as putting her at risk of HIV infection. It was apparent from men such as Juma (age 50), Abraham and Noah (all in discordant relationships) and Ben and Isaiah (whose wives were negative but later got infected) that it was emotionally challenging for those men to hear their wives’ worries or grieving about being put at risk. However, it was rather striking that half of the eight men with sero-discordant wives did not talk about the fear of passing on HIV to them as the primary reason for their reduced interest in sex.

#### Loneliness and isolation from male social spaces

Participants repeatedly stated that living with HIV, and for some, its treatment, had resulted in a loss of social life as they were forced to regulate, or give up completely, a range of leisure activities and interactions with other men, especially the use of conventional male social spaces, as is neatly illustrated by Jeremiah's story. He explained regretfully that:

[Before I fell ill] I used to be with other men; doing work together, moving from place to place and especially in the evening hanging out. But now [due to illness] I have to accept to be like the child who remains to keep the home.

Jeremiah told GES that this ‘isolation for long periods’ was the part of his experience with the illness which he had come to hate most. Although ART was expected to resolve this problem by healing their bodies, freeing them to enter the social arena again, for some men this transformation was not instant. For instance, Jeremiah found the effects of ART rather slow: ‘ … but you have to accept it because I cannot change this condition now, it has to be slowly’. Other men reported that despite ART, they sometimes suffered prolonged illness episodes which continued to intermittently disrupt their social network and the opportunities that this presented. For most participants, this involuntary isolation was both difficult and frustrating to learn and accept.

Although physical recovery due to ART meant that some men were able to restore their company and masculinity with other men, they still faced a dilemma finding the right balance between life on ART and the conventional life style of men in the village. For many men, the ‘rules’ of ART, such as the requirement for reduced smoking and alcohol consumption, both of which most men in the village tend to consume, particularly in the company of others in bars, and adherence to strict and fixed time schedules for swallowing the drugs, caused additional and fundamental lifestyle changes. Drinking beer had been a source of pleasure, a means to overcome worries and/or a social activity to interact with others. But proper adherence to HIV treatment imposed restrictions on alcohol use and discouraged smoking, despite these being considered important activities that usually brought men together. In addition, disclosing one's HIV status/treatment to others exposed one to forms of positive discrimination from colleagues who often advised against alcohol consumption, which further resulted not only in a sense of isolation but also in reduced opportunity to fully enjoy male company, and express and confirm their masculinity.

However, the extent to which men felt their masculinity undermined by social exclusion varied according to the level of disclosure of their HIV status. Men who had disclosed or who believed that the majority of their friends were aware of their infection/treatment, notably Noah and Abraham, found it easier to reject peer pressure to execute normative masculinity behaviour of homo-sociality as a proof of being sufficiently manly. For example, Noah often told GES that he had nothing to disguise when he was with friends, and in case he came under pressure to do something that he felt was not right for his health, he often excused himself by referring to his health/medication condition. Age was another factor that shaped men's sense of self-worth. Those aged over 40 were more self-assured and felt they had nothing much to lose in terms of social lifestyles. Many of them said they had already ‘seen it all, after all’, while others, such as Abraham, argued that their seniority in age ensured that they enjoyed some respect from the village and, therefore, saw no reason to execute other behaviour to express manliness.

### Resuscitated masculinities

Although HIV devastated or seriously threatened men's lives and sense of masculinity, most men found that ART allowed them to turn it around and take control of many aspects of their lives. ART both enabled men to again perform normative ideals of masculinity, and allowed them to re-examine some common norms and develop a different sense of masculinity which they thought was consistent with and safe for their life with HIV.

#### Healthier bodies

All men receiving ART recounted that without it, HIV-related illness rendered them ‘weak and useless’. This reference to uselessness was an admission that many aspects of their social and physical identity as men were significantly threatened and challenged. But for the majority of those who initiated ART, with the exception of instances of side effects, the physical recovery was steady, consequently enabling them to engage in the performance of certain social practices and roles that affirmed their identities as men. For example, when Jeremiah remarked ‘[ … ] you wait: when the body improves you will soon see me at the trading centre with other men’, he was not only referring to the importance of physical strength but also valued it as an essential element for enjoying male company, thus restoring the social body.

Participants also indicated that HIV infection and treatment had led them to become more attentive to their health in ways that they had never been before. In particular, men receiving treatment stated that they always paid close attention to the body changes they experienced, especially regarding changes in weight or strength, and were very sensitive to how their lifestyles might impact on their ART and health. They admitted that their increased concern with health was exceptional and similar to that of women, but argued that it was vital for men, too, to care about their health. Abraham, Noah and Salim (age 45), for example, spoke very confidently about their ability to counter any questioning of their changed lifestyles or male stigmatisation, since they believed that ART was more crucial than anything else for prolonging their lives. Others, such as Isaac, Jeremiah and Salim, argued that norms such as smoking and drinking had become less important to their social identities, maintaining that, instead, what mattered first and foremost was *akijar* (life) and *angaleu* (health), followed by the opportunity afforded by ART for them to perform essential masculine roles in their families. In this way, men on ART drew on their medication regime to rationalise their rejection of some of the predominant but potentially harmful reputational masculine practices.

However, men's readiness to adopt less conventional lifestyles for their health varied with age. Men aged over about 40 appeared less concerned with maintaining conventional masculine lifestyles. Nevertheless, generally, men receiving ART appeared far less anxious about the prospects of embracing less conventional practices than men who had failed to initiate or dropped treatment, who remained concerned to fulfil normative reputational behaviour such as drinking and smoking.

Some men discussed disclosure of their HIV status to their children. Most stated that although they found this difficult, it was important to disclose to the older children, in order for them to know the risks. Juma said, ‘I told the old children (teenagers) so that they can know how I got it [HIV]’.

#### Fatherhood

Without exception, children were valued as a man's most important legacy, the best way to ensure they will be remembered as having once existed in their clans. For some men, like Isaac, HIV infection had frustrated their attempts to have children. However, by making it possible to have HIV-free children through reduced viral load and PMTCT, HIV treatment gave hope for and/or enabled some men to have offspring or add children, thus fulfilling social and individual expectations. In particular, men such as Noah, who said he ‘had not yet had enough children’, or those who had none at the time they tested positive, such as Isaac, spoke very positively about how ART enabled them to have the children they desired. As we saw in Isaac's story, when his wife lost each of their four unborn babies, suspected to be AIDS related deaths, his relatives and friends mockingly told him to stop wasting time and admit he would never have children. ‘As a man, I was distressed by the possibility of leaving the world without a child. I knew there was something lacking in my life because nothing would show that I was also a man in our family’. But with ART, Isaac's paternal dream was salvaged when his wife gave birth to an HIV-negative baby, transforming his image and endearing him to his family again. Such success confirmed masculine respectability as ‘complete’ man.

Although some men knew that the prevention of vertical transmission was mostly dependent on the mother's HIV status, and whether she received nevirapine during child birth, most believed and talked of how their own medication contributed substantially to this outcome. However, most men on ART portrayed their decisions to have children as being under the regulation and control of the health workers, rather than being dependent on their own free will. Men like Isaac felt more confident in justifying to the public, and to GES, their decisions to have children by narrating that they did it ‘after receiving permission from health workers’. But those who did not get permission or had never discussed their plans with counsellors, such as Leo (age 28), expressed a sense of guilt and uncertainty as to whether they were doing the right thing, undermining their sense of independence. In spite of the belief that with ART they could now have HIV-free children, the majority of men reported being cautious about having large numbers of children since they believed that they were poor, and had a shorter lifespan to bring them up compared to other men.

#### Gender equality and fidelity in marriage

In response to changes in their health and/or the demands of ART, many men reviewed their previous high-risk ways and adopted a more reflexive and flexible approach to demonstrating their masculinity. In particular, there was increased tolerance to gender equality in households and rejection of extramarital relationships, saying that their new lifestyles were in fact the ‘appropriate’ ways to live as men.

The majority of men believed and regretted that their infection was a result of their own risky sexual behaviour, rather than that of their wives, and spoke about the need for responsible sexual behaviour. In this regard, most of them talked of no longer being tempted by infidelity. Although some, like Jeremiah, were still physically incapable of sex, and others, like Isaiah, had little confidence that new partners would accept them, many men, especially those receiving treatment, simply argued that they had come to realise it was not good practice to have extramarital partners. Noah, Abraham, Mike and Isaac (all on treatment) repeatedly challenged that infidelity was a marker of masculinity, a position that they partly attributed to the counselling they had received. They said that they could no longer afford to express their masculinity through multiple sexual relations because it would expose them to the risk of more drug-resistant HIV virus strains, which would undermine the gains of ART. Thus, for the majority of men on ART, extramarital affairs were now seen as unhealthy to them and their partners, in contrast to their view point prior to diagnosis. Nevertheless, many of these men were also aware of the publics’ judgemental views about HIV-positive people's sexual activity, and this may have further discouraged extramarital affairs. Furthermore, some may have underreported involvement in extramarital relationships.

HIV also appeared to modify men's perception of gender relations within the household. Although some had experienced a masculine crisis when their dominance in the home was undermined by illness and over-reliance on other family members, many men living with HIV had developed a tolerance towards equality with their wives. From both the interviews and the informal observations of domestic relations, the majority of men with HIV appeared to endorse equality and were less inclined to use domestic violence and aggression than in the past as a way to assert their manliness. This appeared to arise from their realisation that, in fact, their wives and children played crucial support roles in their care and therefore a confrontational life at home would be of no benefit to their treatment efforts. However, this tendency towards egalitarian domestic relations might also be explained by the men's sense of guilt that they had brought HIV infection home. Given this belief, men did not wish to frequently antagonise their wives whom they felt had tolerated them despite their ‘weaknesses’. Many men like Noah, Juma, Abraham and Silver (age 50) emphasised the importance of being tolerant and caring towards all members of the family including one's children. Some categorically answered ‘no’ to GES's question whether they found their new lifestyles and forms of masculinity to be detrimental, constraining and less manly.

## Discussion

The impact of chronic illnesses on masculinity has been assessed for several conditions (e.g. Charmaz 1994; Emslie & Hunt 2009; Emslie *et al*. 2006; Hilton, Emslie, Hunt, Chapple & Ziebland 2009) but much less for HIV, particularly in SSA. Studies that have focused on identity (re)construction following a chronic illness, such as cancer, coronary heart disease and depression, have suggested that the diagnosis not only impacts on someone's state of health, but often also directly intervenes in (re)shaping his or her subjectivity and identity (Emslie *et al*. 2007; Emslie & Hunt 2009; Livneh, Lott & Antanak 2004).

Our data support these studies. In Mam-Kiror, eastern Uganda, HIV diagnosis caused extreme anxiety and uncertainty with regard to masculine identity. For most men the experience of AIDS dented their masculine identity, while this was partly resuscitated following ART. However, the balance between eroded and restored masculinity varied between men in relation to their history of treatment, age, physical state of health, family circumstances and economic circumstances (Siu, Wight & Seeley 2012). We found there is often a tension between the notion of being able to lead a normal life and the requirement to follow medical advice, because being normal means not being ill (Gregory 2005). This parallels findings on the invasive nature of ART for Italian construction workers: its powerful effect may extend life but it can also shatter the person's ‘life world’ and force a redefinition of the self, of one's possibilities and priorities, which can greatly influence adherence to treatment (Alcano 2009).

We found that prior to illness and diagnosis of HIV infection, men endorsed the conventional perceptions of what it means to be a man, within the value systems of both ‘reputation’ and ‘respectability’ (Wilson 1969), notably engagement in multiple sexual relationships, authority and control over family, having children and providing for family and confidence in the use of social and public spaces. However, following HIV illness, men's ability to fulfil most of their obligations, and express their masculinity through previously internalised ideals, was significantly threatened. As found with another chronic illness, such as prostate cancer (O'Brien *et al*. 2007), the challenges to men's masculine identities tended to depend on age, treatment and the extent to which their illness interfered with their social practices.

Our study found that, irrespective of treatment, many men reported a loss of authority over wives and children, either because they had lost the physical strength to dominate them or had failed to provide for them by not working, and instead depended on wives and children for care and support. This resulted in a domestic role reversal, with wives, and even children, becoming more dominant in decision-making, and often disrespectful. Similarly in the UK, black men's loss of earning power due to HIV affected household gender dynamics, with men being particularly concerned about their loss of control and authority in domestic decision-making (Doyal, Anderson & Paparini 2009). Thus, while improvements in health following ART may increase men's optimism about the future, this may be undermined by concerns about being unable to meet strongly gendered expectations in relation to family and work (Fitzgerald, Collumbien & Hosegood 2010), which undermines their respectability. We found that men who engaged in hard physical work again to earn an income following treatment were concerned about exhausting the health gains of ART, while the positive discrimination and sympathies from colleagues made them feel that their masculine ideals of strength and hard work were being undermined (Siu *et al*. 2012).

HIV infection also significantly threatened the enactment of masculinity through sexual achievement, and fathering children, although men were aware that ART improved the chance to have a child. Their accounts seem to reflect both the message they had received from their counsellors to conserve energy and prevent onward spread of HIV and their own realistic evaluation of their ability and chances to get partners, given their perceived unattractive physical appearance and sexual history. In a South African township it was found that most HIV-positive men attempted to prove their masculinity through normative masculine behaviour, such as fathering children (Sakhumzi 2008). However, in our study the rules of ART, such as reducing (risky) sexual activity (Allen, Mbonye, Seeley, Birungi, Wolff, Coutinho *et al*. 2011), and the widespread social sanctions against the sexual activity of known HIV-positive people meant that men were less confident about their sexual abilities, or expressed them discreetly, undermining their reputation before peers and sexual partners. Furthermore, some suffered low interest to engage in sex, and associated it with the ART drugs they were taking. Similar findings have recently been reported in Uganda (Allen *et al*. 2011). Notably, erectile dysfunction has been reported to be fairly common among men experiencing a range of chronic illnesses, especially prostate cancer, hypertension and diabetes, and is associated with a poor quality of life as it affects the emotional, social, sexual and recreational aspects of life (Idung, Abasiubong, Ukott, Udoh & Unadike 2012). However, men infected with HIV have been found to have a higher prevalence of erectile dysfunction than those without HIV (Zona, Guaraldi, Luzi, Beggi, Santi, Stentarelli, *et al*. 2012), and it tends to be associated with increasing age, longer duration of HIV infection and longer usage of ART (Crum-Cianflone, Bavaro, Hale, Amling, Truett, Brandt, *et al*. 2007).

Generally, the younger men tended to have more fragile masculine identities, lacking confidence that they could build and support families, and construct a sensible identity among their peers and the wider society, significantly undermining both their reputation and respectability, while older men were more assured and felt they would lose little by dispensing with certain masculine norms, as found in South Africa (Fitzgerald *et al*. 2010). These findings support previous research on masculinity and chronic diseases which suggest that older men tend to accept loss of aspects of their masculinity, such as sexual functioning and termination of employment, relatively easily compared to younger men because such losses can be attributed to ageing (Cameron & Bernardes 1998).

Nonetheless, our data also provide valuable insights into how HIV and ART may provide some opportunities for men to re-examine some of the norms they had taken for granted and develop a different sense of masculine identity, largely a positive one, which they felt was consistent with, and safer, for their life with ART. Many men on treatment reported an increased tolerance to domestic gender equality and rejection of extramarital relationships, and argued that the new lifestyle was in fact the more appropriate way to act as a man, in the process restoring their respectability both at home and in society (Siu *et al*. 2013). For some men, HIV infection and treatment prompted more attention to health, particularly those on treatment who wondered how practicing conventional reputational masculine ideals might affect it. In the UK, it has been found that some men with chronic diseases are prepared to lose previously valued aspects of masculinity if they make them more vulnerable to the disease; instead they seek alternative social practices to affirm masculinity (O'Brien *et al*. 2007). It has been suggested that displays of competence in hegemonic health-related domains can produce masculine capital which can be used to compensate for non-masculine behaviour in other domains (De Visser, Smith & Mcdonnell 2009).

## Conclusion

This study highlights the various ways in which the experience of HIV and ART modifies masculine identity and men's gendered roles within the household. We found that HIV infection and/or its treatment had varied influences on enactment of both reputational and respectable masculinities. Overall, men living with HIV and on ART underwent challenging experiences with regard to their expression of previously valued performances of masculinity, both at home and in the wider society. Men lost authority and power within the domestic sphere and their relationships with their households were complex and largely disempowering. More widely, some men endured physical isolation from peers and social sanctioning of their sexual activity, substantially hindering them from expressing their conventional masculine identities based on reputation. However, within this context, opportunities arose for men, especially those receiving ART, to review previously risky aspects of masculinity and adopt a more reflexive, positive and flexible approach to demonstrating their masculinity. HIV support agencies need to pay attention to the way HIV and ART influence men's perception of their masculinity and support them to overcome anxieties about dented or eroded masculinity, while building on the positive ways in which treatment restores masculinity to support men's adherence to HIV treatment. In particular, there is a need to support men's engagement in productive activities that generate income so that men can regain their provider roles following ART and thereby restore their respectability. Future research should explore how HIV and ART change men's paternal roles and the transmission of male sexual identity to their sons.
